# A multidimensional clinical prediction model for early screening of recurrent spontaneous abortion: integrating coagulation, immune, and endocrine markers

**DOI:** 10.3389/fimmu.2026.1774359

**Published:** 2026-03-06

**Authors:** Daqi Chen, Anping Liu, Xiaoxia Wang, Xiaoming Liu, Wenjie Liang, Linsheng Luo, Hua Nie, Xingming Zhong

**Affiliations:** 1 National Key Laboratory of Male Reproductive Genetics, Guangzhou, China; 2 Reproductive Immunology of Guangdong Provincial Reproductive Science Institute, Guangzhou, China; 3 School of Mechanical and Electrical Engineering, Guangzhou University, Guangzhou, Guangdong, China

**Keywords:** feature selection, machine learning, multidimensional, recurrent spontaneous abortion, screening model, TabPFN

## Abstract

**Objective:**

Recurrent spontaneous abortion (RSA) affects 0.5%–2.5% of fertile couples and arises from complex, interacting thrombotic, immune, coagulation, endocrine–metabolic, and demographic factors. However, current early risk stratification in routine practice remains insufficient for population-level screening. We aimed to develop an accurate, low-cost, and clinically feasible early screening model for identifying women at high risk of RSA using routinely available clinical biomarkers.

**Methods:**

This retrospective study enrolled women attending Guangdong Reproductive Hospital between 1 January 2020 and 31 December 2024. Among 1226 screened individuals, 285 met eligibility criteria and were included (181 RSA patients and 104 healthy controls). Demographic and laboratory variables were extracted from electronic medical records and structured follow-up. Ten classical machine-learning algorithms and a Transformer-based tabular model (TabPFN) were trained and compared. Class imbalance was handled using the synthetic minority oversampling technique (SMOTE). Model robustness was evaluated using 5-fold cross-validation. Biological-domain contributions were quantified through ablation analysis. Feature selection was optimized using recursive feature elimination with random forest (RFE-RF), and interpretability was assessed via SHAP.

**Results:**

The TabPFN Multidimensional model integrating features across six clinical domains achieved the best discriminative performance for RSA risk prediction (ROC–AUC = 0.927, 95% CI 0.891–0.947), outperforming all comparator algorithms. Domain ablation showed that removing any single biological category reduced performance, supporting the complementary value of multidimensional clinical integration. Acquired thrombophilia markers provided the strongest predictive contribution, followed by hereditary thrombophilia, immune indices, coagulation parameters, endocrine–metabolic variables, and demographic factors. A parsimonious six-biomarker model—anti-phosphatidylserine/prothrombin antibodies (aPS/PT), protein C (PC), antinuclear antibodies (ANA), antithrombin III (AT-III), thrombin time (TT), and body mass index (BMI)—retained high discrimination (AUC = 0.925) with 83% accuracy, supporting a pragmatic and cost-effective screening strategy. SHAP analysis identified elevated aPS/PT, ANA positivity, reduced AT-III activity, and prolonged TT as the most influential predictors, implicating thrombo-immune dysregulation as a central mechanism associated with RSA.

**Conclusion:**

A Transformer-based tabular model using six routinely measured, low-cost biomarkers enable accurate, interpretable, and scalable early screening for RSA risk, with potential utility in resource-limited settings to facilitate timely referral and targeted preventive management.

## Introduction

Recurrent Spontaneous Abortion (RSA), defined as two or more consecutive spontaneous pregnancy losses before 28 weeks of gestation with the same sexual partner, is one of the most prevalent complications in early pregnancy. Globally, it affects an estimated 0.5%–2.5% of women of reproductive age, accounting for approximately 23 million pregnancy failures annually (1). RSA is a heterogeneous condition influenced by multiple risk factors, including maternal age, prior history of pregnancy loss, coagulation abnormalities, immune dysregulation, endocrine dysfunction, genetic predispositions, anatomical abnormalities of the reproductive tract, environmental exposures, and psychosocial factors, which interact across multiple physiological systems. Despite extensive diagnostic evaluations, 50–70% of cases remain unexplained (2), underscoring the complexity and multifactorial nature of the disorder. The heterogeneity of its definition and pathogenesis has hindered timely diagnosis and effective intervention. Therefore, the development of a comprehensive, data-driven screening model that integrates diverse clinical features is urgently needed to identify individuals at high risk and enable early, personalized management strategies.

Attempts have been made to estimate the likelihood of recurrent pregnancy loss using mathematical models. In the mid-20th century, Malpas and Eastman proposed formulas to calculate the risk of subsequent miscarriage based on the number of previous pregnancy losses (3). More recently, several studies have identified predictive factors and developed models to estimate live birth outcomes among patients with RSA (4–10). However, most of these models rely on logistic regression and primarily incorporate maternal age and prior pregnancy loss history. While these demographic factors are important, they fail to capture the active physiological dysregulations driving the pathology.

Pathophysiologically, immune dysregulation and pre-thrombotic states are considered the most common maternal etiologies, while adverse lifestyle and psychological factors serve as predisposing risks. However, RSA is rarely a single-system defect; rather, it results from “pathogenic crosstalk” between systemic domains. This multifactorial complexity is recognized in the “2025 Chinese Expert Consensus on Clinical Practice for Etiological Screening of Recurrent Spontaneous Abortion” (11), which recommends antiphospholipid antibodies and antinuclear antibodies (ANA) as primary screening items for immune factors. For pre-thrombotic states, it recommends coagulation function, platelet aggregation, and thromboelastography, alongside screening for hereditary thrombophilia (protein C, protein S, and antithrombin III) when necessary. Validating the clinical relevance of these markers, Zhao et al. (12) identified independent risk factors—including lupus anticoagulant (LA), anticardiolipin IgM, anti-phosphatidylserine/prothrombin IgM, anti-dsDNA, arachidonic acid-induced platelet aggregation, and thrombin time (TT)—based on multivariate logistic regression. Their resulting nomogram successfully stratified patients into low- and high-risk groups with significantly different pregnancy loss rates (P< 0.001). The mechanistic rationale for integrating these specific indicators lies in their ability to directly map onto the core pathological axes of RSA. Specifically, the aforementioned hypercoagulability and thrombophilia markers reflect the risk of placental microthrombosis and impaired perfusion; the immunological factors indicate the potential breakdown of maternal-fetal tolerance and autoimmune-mediated vascular injury; while endocrine-metabolic parameters serve as the systemic regulatory background that modulates both immune responses and endometrial receptivity. Capturing the synergistic interplay of these domains—coagulation, immunity, and endocrinology—is essential for accurate risk assessment, yet they are rarely integrated into a single clinical screening tool.

The emergence of artificial intelligence (AI) has introduced novel approaches to predicting RSA (13–15). Notably, Bruno et al. (15) applied support vector machine (SVM) modeling to a dataset of 734 RSA patients incorporating 43 clinical features, achieving a balanced accuracy of 81.86%. Yan et al. (16) developed an enhanced framework based on deep learning, using ResNet-50 and TabNet to fuse ultrasound images with clinical data, achieving an AUC value of 0.853. Additionally, Yan et al. (17) employed a method based on XGBoost to extract radiomics features from multimodal ultrasound, with an AUC value of 0.844. Furthermore, Li et al. (18) combined serum autoantibodies with longitudinal ultrasound parameters to achieve a prediction AUC value of 0.92. These results underscore the value of integrative modeling. However, broad-spectrum models often rely on extensive feature sets that may not be universally available or cost-effective in primary care settings. To bridge the gap between high-dimensional data analysis and routine clinical practice, there is a need for a model that maintains high predictive accuracy while utilizing highly accessible and standardized biomarkers.

Building on this rationale, the present study sought to establish a multidimensional risk assessment model for RSA by integrating demographic, hematological, immunological, and metabolic indicators using modern machine-learning architectures. Unlike approaches relying on high-cost genomic profiling, we focused specifically on routinely available clinical biomarkers to ensure the model’s feasibility and cost-effectiveness. To clarify the relative contribution of each biological domain, we incorporated systematic feature-ablation analyses, while SHapley Additive explanations (SHAP) were applied to provide transparent interpretation of model behavior. Furthermore, we evaluated whether a reduced subset of these clinically accessible biomarkers could approximate the performance of the full integrative model. Through this systemic and interpretable approach, our study aims to advance precision risk stratification and support earlier, individualized management strategies in a resource-efficient manner.

## Materials and methods

### Patient population

Clinical data were retrospectively extracted from electronic medical records of women who had presented to Guangdong Reproductive Hospital for reproductive health evaluation between January 2020 and December 2024. Initially, 859 women were identified as having RSA according to the 2022 American Society for Reproductive Medicine criteria, defined as two or more consecutive spontaneous pregnancy losses confirmed by ultrasound as clinically viable pregnancies that terminated before 20 weeks of gestation. Controls comprised 367 age-matched, healthy women of reproductive age with regular menstrual cycles and at least one prior live birth, who had no history of adverse pregnancy outcomes or co-existing autoimmune or endocrine disorders.

Blood samples were collected under standardized conditions to minimize laboratory variability: baseline hormonal profiles were obtained on days 2–3 of the menstrual cycle, whereas all other laboratory assays were performed during the non-menstrual, non-pregnant phase after an overnight fast of at least eight hours.

Exclusion criteria included: (1) chromosomal abnormalities in either or both partners; (2) anatomical abnormalities of the reproductive tract; (3) conception via assisted reproductive technology; (4) use of hormonal therapies or systemic medications affecting the results; (5) severe systemic diseases (e.g., significant hepatic or renal dysfunction, or untreated metabolic disorders); and (6) incomplete clinical records, defined as more than 20% of key laboratory data missing.

**Figure 1:**
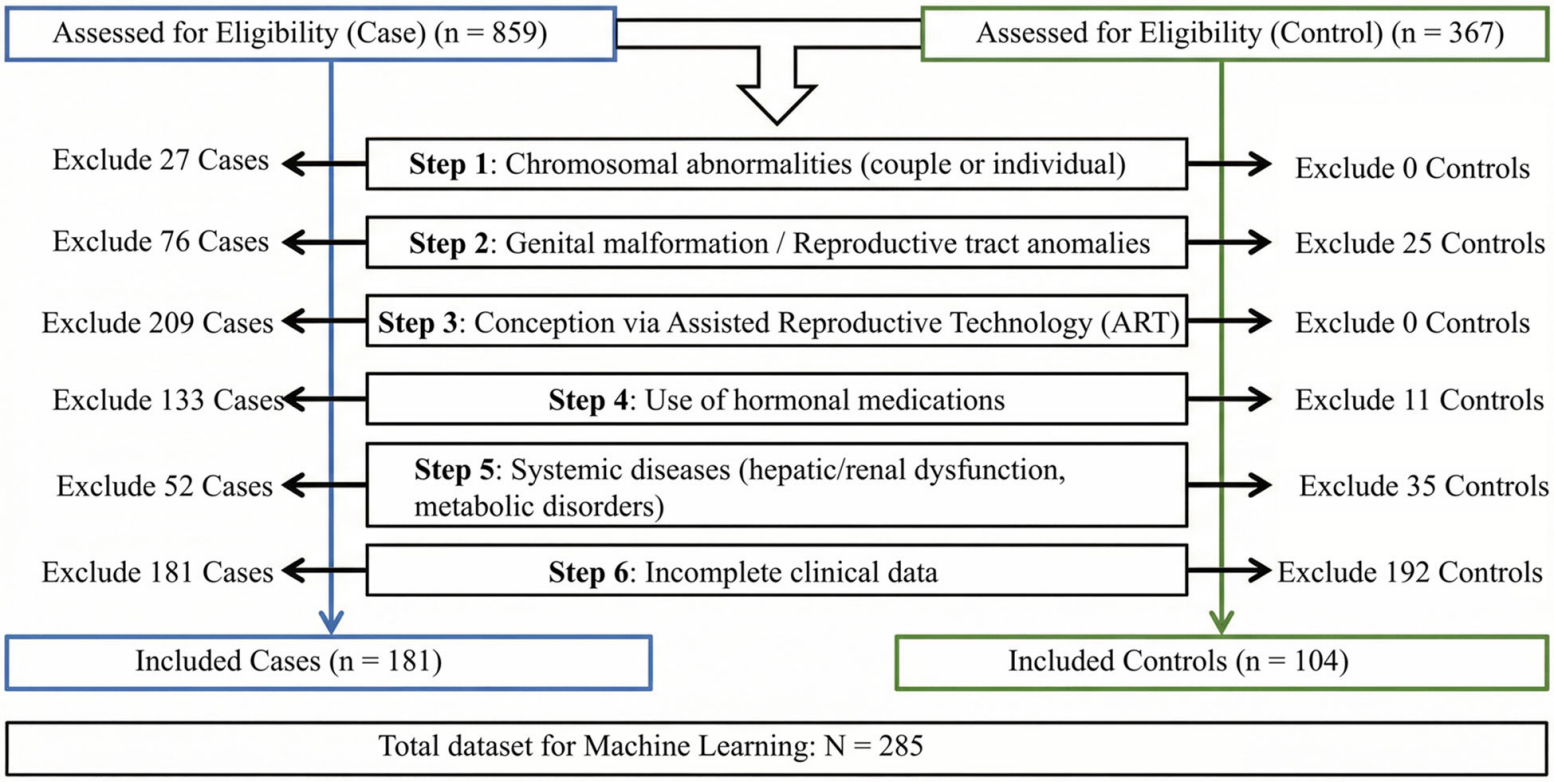
Flowchart of the subject selection process.

After applying these criteria, 687 participants from the RSA pool and 263 from the control pool were excluded. The final dataset comprised 181 women with RSA and 104 healthy controls. The detailed participant flow, including the specific number of exclusions per criterion stratified by group, is summarized in **Figure 1**.

### Study variables

As shown in **Table 1**, study variables were categorized into six clinically relevant domains. Demographic factors included age and body mass index (BMI). Coagulation function comprised TT. Immunological factors included ANA and anti–double-stranded DNA (anti-dsDNA) antibodies. Hereditary thrombophilia markers consisted of antithrombin III (AT-III), protein S (PS), protein C (PC) and the methylenetetrahydrofolate reductase C677T (MTHFR C677T) polymorphism. Acquired thrombophilia markers included anti–phosphatidylserine/prothrombin antibodies (aPS/PT), LA and anticardiolipin antibodies (aCL). Endocrine–metabolic factors consisted of thyroid-stimulating hormone (TSH) and thyroxine (T4).

**Table 1:** Categorization of study variables in this study.

Feature group	Included features
Demographic factors	Age, BMI
Coagulation function	TT
Immunological factors	ANA, dsDNA
Hereditary thrombophilia	AT-III, PS, PC, MTHFR C677T
Acquired thrombophilia	aPS/PT, LA, aCL
Endocrine-metabolic factors	TSH, T4

This classification reflects the clinical and pathophysiological dimensions relevant to miscarriage risk and facilitates structured analysis across demographic, immune, coagulation, thrombophilic and endocrine pathways.

### Data preprocessing

The dataset was randomly divided into training and test sets in an 80:20 ratio. Continuous variables were standardized using Z-score normalization to achieve a mean of 0 and a standard deviation of 1. In accordance with clinical consensus in reproductive medicine, age was categorized into three groups (<30 years, 30–35 years and >35 years). BMI was classified as underweight, normal weight or overweight based on Asian-specific criteria.

Categorical variables were converted using one-hot encoding to prevent the model from inferring artificial ordinal relationships among non-ordinal categories. For binary variables, including the MTHFR C677T polymorphism, one-hot encoding was similarly applied to ensure independent representation of each category and to support robust model performance.

### Model construction and selection of evaluation indicators

To enable a broad methodological comparison, several classical machine learning algorithms were included in the analysis, representing different families of modeling techniques, including linear models, tree-based models, ensemble learning, and support vector machines. The TabPFN algorithm, a deep learning approach based on transformer architecture adapted for tabular data, was also integrated into the study and subjected to model development procedures alongside the other methods.

Model performance was evaluated using a five-fold cross-validation approach. In this method, the dataset was randomly divided into five equal subsets. Four of these subsets were used for training, while the remaining subset was used for testing. All data preprocessing step—including scaling, one-hot encoding, and oversampling—were fitted exclusively on the training folds and subsequently applied to the held-out validation fold to prevent data leakage. This process was repeated five times, with each subset serving once as the test set, which helps minimize potential bias associated with a single data split and enhances the robustness and generalizability of the evaluation results. Model interpretability was performed using SHAP. Given that TabPFN is a transformer-based model without tree structure, SHAP values were computed using the model-agnostic KernelSHAP algorithm. The full training dataset in each cross-validation fold was used as the background distribution. Default sampling parameters provided by the SHAP implementation in TabPFN extensions were used.

A set of standard performance metrics was used to assess model effectiveness, including accuracy, precision, recall, specificity, F1-score, and the area under the receiver operating characteristic curve (ROC-AUC). The 95% confidence intervals (CIs) for AUC were estimated using bootstrap resampling (1,000 iterations with replacement) applied to the AUC values obtained from each of the 5 cross-validation folds, with percentile-based intervals derived from the resampled distributions. The corresponding mathematical definitions of these metrics are presented in (Equations 1–5).

(1) $$\text{Accuracy }=\;\frac{\text{TP}+\text{TN}}{\text{TP}+\text{TN}+\text{FP}+\text{FN}}\;$$

(2) $$\text{Precision }=\;\frac{\text{TP}}{\text{TP}+\text{FP}}$$

(3) $$\text{Recall}=\;\frac{\text{TP}}{\text{TP}+\text{FN}}$$

(4) $$\text{Specificity}=\;\frac{\text{TN}}{\text{TN}+\text{FN}}$$

(5) $$\text{F}1\text{ score}=\;\frac{2\times \text{ Precision}}{\text{Precision}+\text{Recall}}$$

### Feature screening strategy

To develop a population screening strategy for recurrent miscarriage with reduced feature dimensionality yet high predictive performance, the random forest algorithm was employed for feature selection. Random forest evaluates feature importance using the Mean Decrease in Impurity (MDI) metric, which is defined as Equation 6:

(6) $$\text{MDI}\left(\text{f}\right)\;=\;1/{\text{N}}_{\text{trees}}\text{ Σ}\left(\Delta \text{Gini}\_\text{split}\left(\text{f}\right)\right)$$

where N_trees_ denotes the total number of decision trees, and ΔGini_split(f) represents the total reduction in Gini impurity contributed by feature ff across all splitting nodes. This metric quantifies the contribution of each feature to the decrease in uncertainty during tree-based partitioning—features with higher MDI values are considered more informative for predicting the target variable. The feature selection process incorporates strategies such as threshold filtering and recursive feature elimination (RFE), enabling the removal of irrelevant or redundant features while identifying key predictive drivers.

### Ethics

The study was approved by the Ethics Committee of Guangdong Reproductive Hospital (Approval No.: IIT20250801). Due to the retrospective nature of the electronic medical records analysis, the requirement for written informed consent was waived for the majority of participants. However, for the subset of participants enrolled under the Guangdong Medical Science and Technology Research Foundation project (Grant No. A2024004), written informed consent was obtained prior to enrollment.

## Results

### Clinical characteristics of samples

**Table 2** presents a comprehensive comparison of baseline characteristics between the RSA group (n=181) and the healthy control group (n=104), including demographic data, coagulation profiles, immune markers, hereditary and acquired thrombophilia-related factors, and endocrine-metabolic parameters. Statistically significant differences were observed between the two groups in AT-III (P<0.01), T4(P<0.01), TT (P = 0.03), dsDNA (P = 0.01), aCL (P = 0.01), and aPS/PT (P = 0.02). No other variables demonstrated statistically significant differences (P>0.05).

**Table 2:** Clinical characteristics of studied population.

Characteristic	Control group=104	RSA group=181	P value
	(Mean ± SD)	(Mean ± SD)	
**Age**	32.37 ± 4.40	32.60 ± 4.61	0.64
**BMI**	21.94 ± 2.97	22.11 ± 2.49	0.64
**T4**	98.58 ± 29.72	110.76 ± 24.30	<0.01
**TT**	16.67 ± 4.53	17.53 ± 1.26	0.03
	n (%)	n (%)	
LA:			0.12
Positive	1(0.96%)	4(2.21%)	
Negative	103(99.04%)	177(97.79%)	
aCL:			0.01
Positive	2(1.92%)	3(1.66%)	
Negative	102(98.08%)	178(98.34%)	
ANA:			0.12
Positive	4(3.84%)	11(6.08%)	
Negative	100(96.16%)	170(93.92%)	
dsDNA:			0.01
Positive	2(1.92%)	2(1.10%)	
Negative	102(98.08%)	179(98.90%)	
PC:			0.66
Positive	1(0.96%)	8(4.42%)	
Negative	103(99.04%)	173(95.58%)	
PS:			0.68
Positive	4(3.84%)	11(6.08%)	
Negative	100(96.16%)	170(93.92%)	
TSH:			0.63
Positive	19(18.27%)	25(13.81%)	
Negative	84(81.73%)	156(86.19%)	
AT-III:			<0.01
Positive	1(0.96%)	19(10.50%)	
Negative	103(99.04%)	162(89.50%)	
aPS/PT:			0.02
Positive	6(5.77%)	29(16.02%)	
Negative	98(94.23%)	152(83.98%)	
MTHFR C677T:			0.20
Positive	31(29.81%)	62(34.25%)	
Negative	73(70.19%)	119(65.75%)	

Reference ranges for categorical variables were defined as follows:

**Age:** Participant age in years.

**BMI:** Body Mass Index (weight/height², kg/m²).

**T4:** Total Thyroxine levels measured in serum.

**TT:** Total Testosterone levels measured in serum.

LA: values< 1.2 were considered negative, whereas values ≥ 1.2 were classified as positive.

aCL (chemiluminescence immunoassay): values< 12 were considered negative and values ≥ 12 as positive.

ANA and dsDNA (chemiluminescence immunoassay): values< 40 were defined as negative, and values ≥ 40 as positive.

PC: values > 70 were considered within the normal range (negative), while values ≤ 70 indicated a positive result.

PS: values > 42 were defined as negative and values ≤ 42 as positive.

TSH: values< 2.5 mIU/L were considered negative, while values ≥ 2.5 mIU/L were considered positive.

AT-III: values > 83 were classified as negative, and values ≤ 83 as positive.

aPS/PT: values< 20 were considered negative; values > 30 were defined as positive.

MTHFR C677T genotype: the C/C genotype was regarded as negative, whereas C/T and T/T genotypes were classified as positive.

### Development of prediction models

To develop a more robust classification model for RSA based on multidimensional clinical data, ten machine learning models — including decision tree (DT), support vector machine (SVM), logistic regression (LR), k-nearest neighbors (KNN), random forest (RF), gradient boosting (GB), AdaBoost, naïve Bayes (NB), multilayer perceptron (MLP), and extreme gradient boosting (XGB) — as well as a deep learning model based on TabPFN, were implemented and optimized for RSA classification. Each comparative model was configured with appropriate hyperparameters grounded in theoretical principles and empirical experience, with detailed parameter settings provided in **Supplementary Tables 1**, **2**.

**Figure 2:**
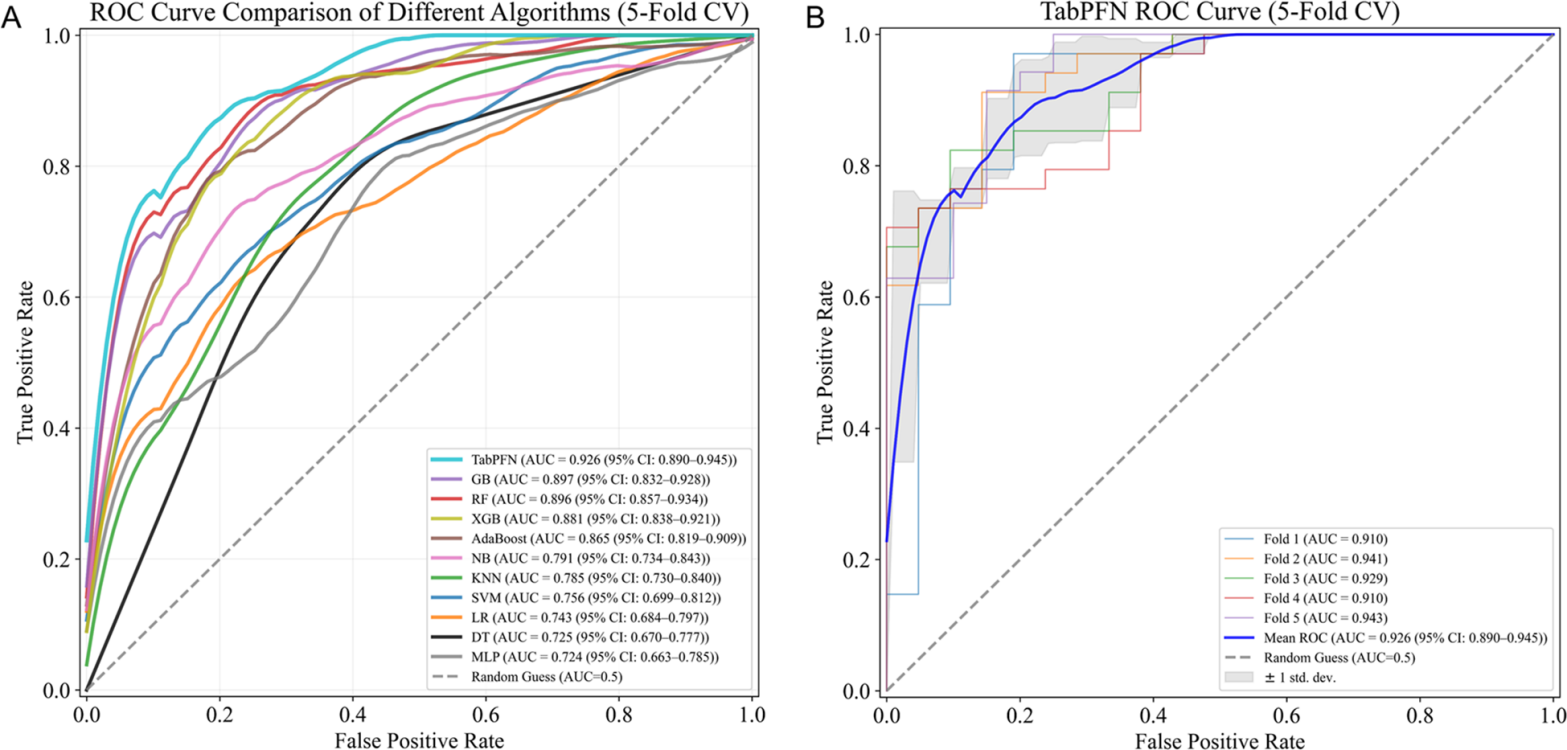
ROC analysis of different models. **(A)** Comparison of ROC curves across models. **(B)** ROC curves for each fold in the five-fold cross-validation of the TabPFN model.

As presented in **Table 3**, the performance of all models was systematically evaluated. The TabPFN model achieved the highest ROC-AUC of 0.926 (95% CI: 0.890–0.945) under five-fold cross-validation. In comparison, the remaining models exhibited relatively lower performance: GB (AUC = 0.897 (95% CI: 0.832–0.928)), RF (AUC = 0.896 (95% CI: 0.857–0.934)), XGB (AUC = 0.881 (95% CI: 0.838–0.921)), AdaBoost (AUC = 0.865 (95% CI: 0.819–0.909)), NB (AUC = 0.791 (95% CI: 0.734–0.843)), KNN (AUC = 0.785 (95% CI: 0.730–0.840)), SVM (AUC = 0.756 (95% CI: 0.699–0.812)), LR (AUC = 0.743 (95% CI: 0.684–0.797)), DT (AUC = 0.725 (95% CI: 0.670–0.777)), MLP (AUC = 0.724 (95% CI: 0.663–0.785)). To provide a comprehensive assessment of model performance, additional evaluation metrics — including accuracy, recall, specificity, and F1 score — were computed. The results indicate that the TabPFN model consistently outperformed all other models across all evaluated metrics. The comparative analysis of ROC-AUC curves among different models, together with the consistency assessment of five-fold cross-validated ROC curves for TabPFN (**Figures 2A, B**), confirm that the model demonstrates superior discriminative capability and excellent generalization stability. Detailed fold-wise performance metrics, including mean and standard deviation for all evaluated metrics across all experiments, are available in the Supplementary Information **Supplementary Table S5**–**S9**.

**Table 3:** Performance comparison of classification algorithms.

Classifier	Accuracy	Precision	Recall	Specificity	F1 score	ROC AUC
MLP	0.676	0.768	0.690	0.655	0.725	0.724
DT	0.734	0.807	0.755	0.701	0.778	0.725
LR	0.680	0.783	0.672	0.694	0.720	0.743
SVM	0.520	0.889	0.263	0.8	0.394	0.756
KNN	0.691	0.831	0.632	0.789	0.716	0.785
NB	0.662	0.812	0.609	0.813	0.696	0.791
AdBoost	0.807	0.847	0.848	0.741	0.847	0.865
XGB	0.821	0.840	0.883	0.721	0.860	0.881
RF	0.815	0.846	0.860	0.741	0.852	0.896
GB	**0.840**	0.861	**0.889**	0.76	**0.874**	0.897
**TabPFN**	0.826	**0.904**	0.804	**0.856**	0.850	**0.926**

The best performance in each metric is highlighted in bold.

### Data augmentation

To address the potential impact of class imbalance on model performance, we evaluated the effectiveness of synthetic minority over-sampling technique (SMOTE) in enhancing data representation. A comparative analysis was conducted between the baseline TabPFN model (Base model) and the model incorporating SMOTE-augmented data (Aug model). Following SMOTE implementation, the dataset was expanded from 285 to 362 samples, resulting in a more balanced feature space distribution (**Supplementary Figure 1**). As demonstrated in **Table 4**, all performance metrics were substantially improved after SMOTE implementation. Sensitivity has improved the most. Regarding discriminative ability, imporving ROC-AUC to 0.927 (95% CI: 0.891–0.947). Rigorous validation through generalization gap analysis (0.006, **Supplementary Table 3**) and stable SHAP importance rankings (**Supplementary Figure 2**) confirmed that the model captures intrinsic biological patterns without overfitting to synthetic artifacts, maintaining high generalizability to real-world data.

**Table 4:** The effect of the SMOTE data augmentation.

Classifier	Accuracy	Precision	Sensitivity	Specificity	F1 Score	ROC-AUC
Base model	0.826	0.904	0.804	0.856	0.850	0.926
Aug model	0.846	0.908	0.840	0.858	0.872	0.927

### Feature group ablation experiments

To systematically evaluate the contribution of each feature category to model performance, we performed a feature group ablation analysis by individually excluding each of the six predefined categories—demographic, coagulation function, immunological, hereditary thrombophilia, acquired thrombophilia, and endocrine/metabolomic features—and comparing the resulting models to the baseline model that incorporated all six categories. Results are summarized in **Table 5**, notably, the exclusion of acquired thrombophilia-related features (aPS/PT, LA, aCL) led to the most significant decline: accuracy dropped from 0.846 to 0.758, and ROC-AUC decreased from 0.927 to 0.856. Furthermore, the exclusion of either hereditary thrombophilia or immunological features also resulted in notable decreases in ROC-AUC. The less influential ones are Coagulation (TT), endocrinary-metabolic (TSH, T4), and Demographics (age, BMI).

**Table 5:** Ablation study of six key physiological dimensions features sets.

Features	Accuracy	Precision	Recall	Specificity	F1 Score	ROC AUC
ALL	0.846	0.908	0.840	0.858	0.872	0.927
Without A	0.758	0.860	0.732	0.800	0.790	0.856
Without H	0.756	0.846	0.742	0.768	0.790	0.862
Without I	0.810	0.876	0.812	0.808	0.842	0.876
Without C	0.820	0.896	0.806	0.846	0.848	0.914
Without D	0.844	0.904	0.840	0.846	0.870	0.922
Without E	0.844	0.892	0.852	0.828	0.870	0.922

A, acquired thrombophilia features; I, immunological features; H, hereditary thrombophilia features; C, Coagulation features; D, Demographic features; E, endocrine-metabolic features.

### Feature selection experiment

Although the multidimensional model achieved high predictive accuracy and highlighted the central role of thrombophilia-related features, its clinical utility is limited by the complexity and cost of data acquisition. Thus, we applied RFE to distill the model into a parsimonious panel of key predictors, enabling a cost-effective and readily implementable screening strategy for early identification of women at high risk of RSA. Feature importance (MDI) was estimated within each of the five cross-validation folds using only the corresponding training data, ensuring that validation samples did not influence feature selection. MDI values are presented in **Supplementary Figures 3A–E**, with the averaged importance across folds shown in **Supplementary Figure 3F**. In each iteration, the feature with the lowest MDI value was systematically removed, the model was retrained, and corresponding performance changes were recorded (see **Table 6**, and **Supplementary Figure 4**). Experimental results revealed a significant non-linear relationship between the number of features and model performance. The ROC-AUC improved steadily as the number of features increased from 1 to 6, achieving an optimal value of 0.925 (95% CI: 0.887–0.942) with the six-feature combination. Beyond this point, further inclusion of features yielded minimal improvement in model performance. Ultimately, the six most robust predictors identified were aPS/PT, PC, ANA, AT-III, TT, and BMI.

**Table 6:** Model performance during RFE.

Number of feature	Accuracy	Precision	Recall	Specificity	F1 Score	OC AUC
14	0.846	0.908	0.840	0.858	0.872	0.927
13	0.84	0.901	0.836	0.847	0.866	0.926
12	0.84	0.9	0.836	0.846	0.865	0.917
11	0.833	0.899	0.825	0.847	0.859	0.923
10	0.836	0.894	0.836	0.837	0.863	0.924
9	0.836	0.91	0.819	0.866	0.86	0.921
8	0.836	0.901	0.83	0.846	0.862	0.919
7	0.847	0.901	0.848	0.846	0.872	0.925
6	0.833	0.911	0.813	0.865	0.856	0.925
5	0.829	0.904	0.813	0.855	0.854	0.91
4	0.786	0.91	0.731	0.876	0.808	0.911
3	0.789	0.895	0.755	0.847	0.815	0.906
2	0.753	0.871	0.708	0.827	0.78	0.867
1	0.698	0.921	0.567	0.913	0.699	0.796

### Risk stratification based on the optimal clinical cut-off

To enhance the translational utility of the six-biomarker model, we determined the optimal decision threshold by maximizing the Youden index on the ROC curve. The peak Youden index (0.678 predicted probability) was adopted as the clinical cut-off. Participants were accordingly classified into a high-risk group (> 0.678) and a low-risk group (≤ 0.678). As shown in **Table 7**, the subsequent miscarriage rate was 88% in the high-risk stratum versus 10% in the low-risk stratum (P< 0.001), confirming that the selected threshold effectively discriminates clinically meaningful risk categories.

**Table 7:** Risk stratification based on the optimal clinical cut-off value.

	Abortion rate	P value
High risk	0.88	<0.001
Low risk	0.10	

### Model performance in clinically significant subgroups

To further evaluate the reliability and clinical practicability of the model in risk stratification, subgroup analysis was conducted on the population with higher baseline risk, including elderly mothers (age>35) and those with a higher body mass index (BMI>24). As shown in **Table 8**, the model maintained excellent discrimination ability in all key subgroups. In the high age subgroup, the accuracy of the model was 0.881, and the ROC-AUC was 0.933. Similarly, in the high BMI subgroup, the model performed well, with an accuracy of 0.943 and a ROC-AUC of 0.947, both higher than the data from the overall population analysis (0.925), indicating that in metabolically susceptible individuals, the model has a stronger ability to distinguish risks.

**Table 8:** The model performance of different population subgroups.

Classifier	Accuracy	Precision	Sensitivity	Specificity	F1 score	ROC-AUC
All people	0.833	0.911	0.813	0.865	0.856	0.925
High Age	0.881	0.919	0.913	0.843	0.915	0.933
High BMI	0.943	0.951	0.921	0.936	0.935	0.947

### Model interpretation

**Figure 3:**
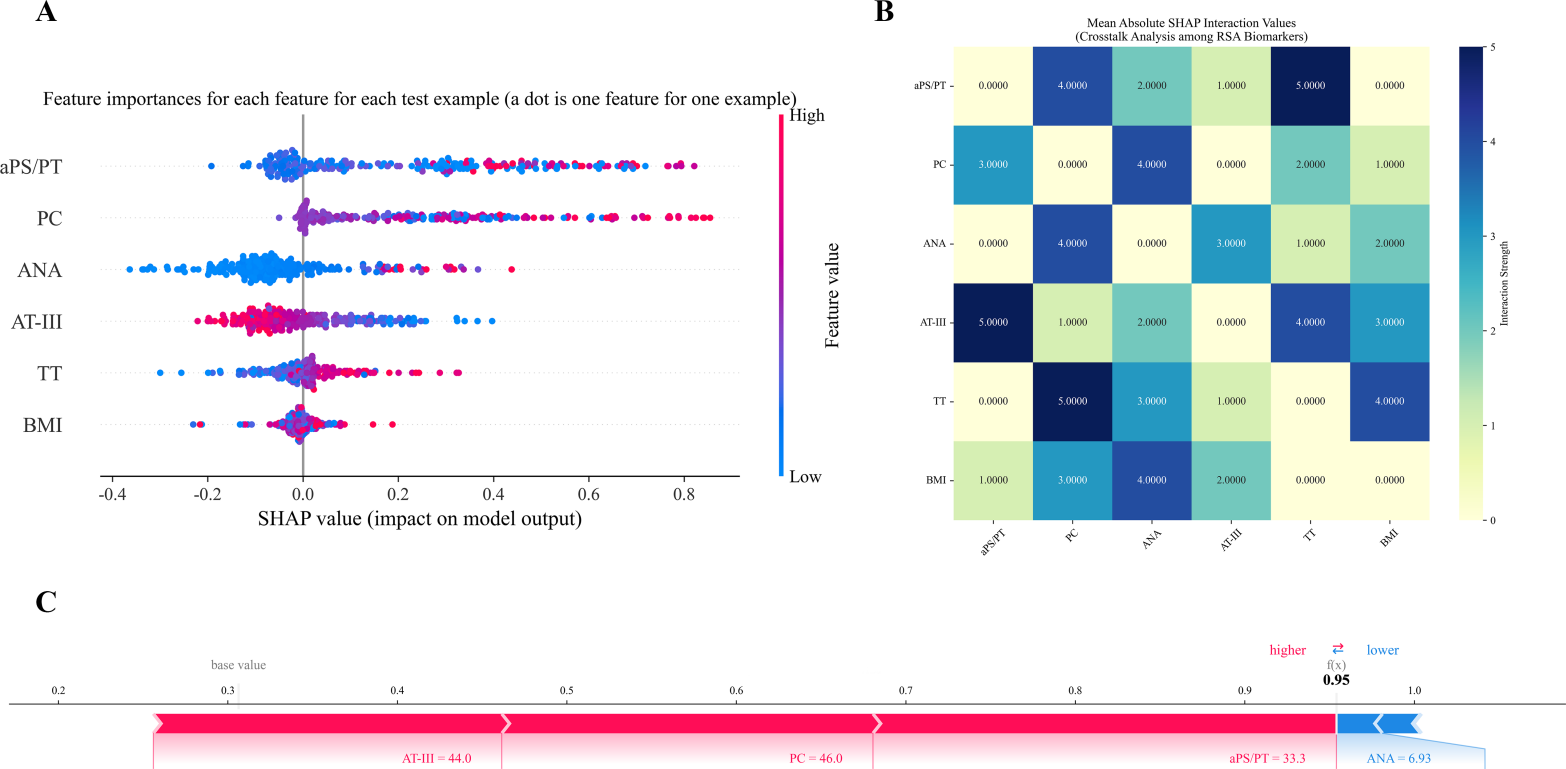
SHAP-based analysis of feature importance and interactions in RSA risk prediction. **(A)** Feature importance ranking of six core biomarkers. **(B)** SHAP interaction effects between key coagulation-immune biomarkers. **(C)** SHAP Force plot for an individual sample**’**s prediction, explaining how different features contribute to deviating the prediction from the base value (baseline 0.306 → 0.9).

To provide an intuitive interpretation of the selected variables, SHAP analysis was employed to visualize the contribution of each feature to RSA risk prediction in the model (19). As shown in **Figures 3A, the** six most influential predictors—aPS/PT, PC, ANA, AT-III, TT, and BMI—were identified. Each dot represents an individual patient’s data point, with color intensity reflecting feature value magnitude: red indicates high-risk values and blue indicates low-risk values. Higher aPS/PT and ANA levels, reduced AT-III activity, prolonged TT, and elevated BMI corresponded to increased SHAP values, indicating a greater likelihood of RSA. Importantly, this approach highlights that even when individual biomarkers remain within clinically “normal” ranges, their combined patterns can stratify patients into higher-risk categories. Such insights not only align with established clinical understanding but also provide a rational basis for early intervention.

To further investigate the complex dependencies among these biomarkers, a SHAP interaction heatmap was generated (**Figure 3B**). This visualization captures the strength of pairwise interactions, revealing that RSA risk is driven by thrombo-immune-metabolic crosstalk rather than isolated abnormalities. Notably, combinations such as aPS/PT+TT, AT-III+aPS/PT, and TT+PC exhibited strong synergistic effects. These patterns suggest that the convergence of acquired or inherited thrombophilia with coagulation dysfunction creates a potent “double-hit” state of hypercoagulability, which the model successfully leverages for risk stratification.

Representative SHAP force plots on the probability scale further demonstrated individual contributions (**Figure 3C**). In a high-risk patient (predicted probability = 0.95), AT-III=44, PC = 46, and aPS/PT=31.3 were identified by SHAP as the primary contributors driving the high-risk prediction.

To ensure the reliability of model interpretations, we assessed the stability of SHAP-based feature attributions across the five cross-validation folds (**Supplementary Table 4**). Feature importance rankings showed high consistency across folds (Kendall’s W = 0.95), and the top predictive biomarkers exhibited low variability in their mean absolute SHAP values (coefficient of variation< 0.15).

### Development of visual tools for clinical diagnosis

**Figure 4:**
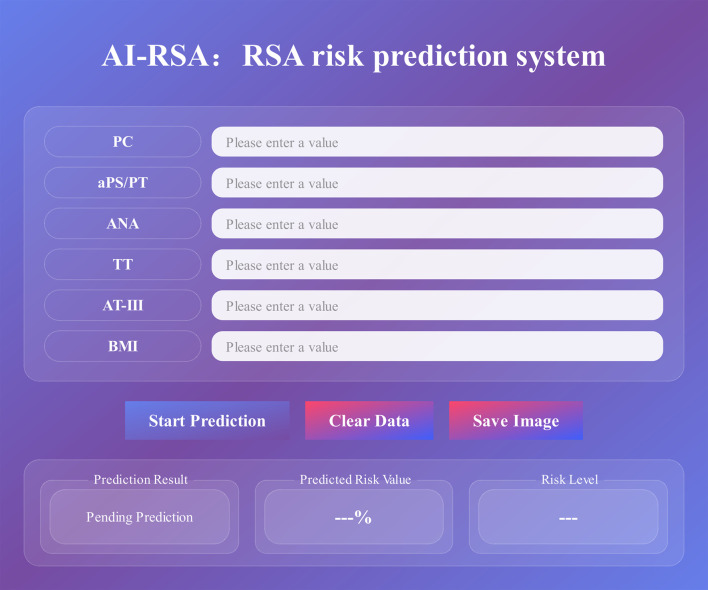
Interface of AI-RSA prediction platform.

To promote the clinical translation and extensive validation of the model, we have developed an interactive platform for RSA risk stratification based on the optimized AI-RSA (**Figure 4**). Clinicians can input the six clinical parameters to obtain a probability score for miscarriage risk. On a standard office PC (Intel Core i5/i7 CPU) without GPU acceleration, the model inference latency is approximately 5 ms, allowing for seamless integration into real-time prenatal consultations (**Supplementary Figure 5**). Detailed descriptions of the intended clinical workflow, decision support protocols, and guardrails to mitigate the impact of false positives are provided in **Supplementary Figure 1**.

## Discussion

RSA remains a significant clinical challenge, affecting up to 5% of reproductive-aged women and often resulting in promoted diagnostic uncertainty and empirical treatment. Although several studies have attempted to model the risk of RSA (13–15), these efforts are often constrained by narrow biological scope, limited external validation, and inadequate model interpretability. Approaches focusing on single biological pathways frequently overlook critical thrombo-immunological interactions, resulting in suboptimal predictive performance (typically modest AUCs) and limiting clinical utility. Meanwhile, current clinical practice relies heavily on subjective assessment or rigid guideline-based panels, which lack sensitivity and contribute to high false-negative rates (20, 21).

To bridge this gap, we developed a high-performance, interpretable risk prediction model for RSA using the TabPFN algorithm trained on a comprehensive set of multidimensional clinical features spanning demographic, coagulation, immunological, and thrombophilia-related domains. Our full model achieved an ROC-AUC of 0.927 (95% CI: 0.891–0.947), demonstrating strong discriminative ability for early risk stratification. This performance matches the robust benchmarks set by recent state-of-the-art models utilizing multimodal ultrasound or extensive laboratory panels (15–18). Complementing these comprehensive approaches, our work further emphasizes clinical accessibility by distilling predictive complexity into a streamlined workflow (**Table 9**). Critically, through systematic ablation studies and SHAP-based interpretability analysis, we identified a core set of six routinely available variables—aPS/PT, PC, ANA, AT-III, TT, and BMI—that together form a parsimonious yet highly accurate predictive panel (AUC ≈ 0.925). This streamlined model retains near-equivalent performance to the full feature set while a cost-effective alternative suitable for large-scale screening. Collectively, our framework represents a step toward precision screening in RSA, offering a scalable, evidence-based tool to support timely and individualized care.

**Table 9:** Machine learning models for RSA prediction: feature, performance and clinical feasibility comparison.

	Model	Feature coverage	Performance metrics	Clinical feasibility
Ours	TabPFN (Transformer)	Six clinical routine indicators	AUC: 0.925	Low cost, no complex equipment, suitable for population screening
Bruno et al. (2020) (15)	SVM	18–43 clinical	Acc: 90.24%	Many variables, requiring comprehensive screening
Yan et al. (2024a) (16)	ResNet50 + TabNet	Gray-scale ultrasound images + clinical data	AUC: 0.853	Requiring expert image collection and offline analysis
Yan et al. (2024b) (17)	XGBoost	Multimodal ultrasound imaging biomarker (SWE + gray-scale) + clinical data	AUC: 0.844	Requiring SWE equipment and complex processing
Li et al. (2026) (18)	LR	Autoantibodies + Ultrasound parameters	AUC: 0.92	Requiring multiple transvaginal ultrasound monitoring

Our findings identify acquired thrombophilia as the most influential determinant of RSA, reflecting the molecular and immunological drivers of a prethrombotic state. This domain integrates key autoimmune-mediated coagulopathies, particularly antiphospholipid syndrome (APS), which compromise placental perfusion through microthrombosis, endothelial injury, and local inflammation, ultimately leading to irreversible placental dysfunction. In the laboratory evaluation of acquired thrombophilia, the antiphospholipid antibody (aPL) profile constitutes the cornerstone. According to current international criteria, diagnostic markers for APS include aCL, anti-β_2_-glycoprotein I antibodies (anti-β_2_GPI), and LA. However, recent studies have highlighted aPS/PT as non-criteria aPLs that are markedly elevated in obstetric APS and strongly associated with adverse pregnancy outcomes, including preterm birth, fetal growth restriction, preeclampsia, and RSA. Li et al. (12) demonstrated that aCL positivity is a significant risk factor for subsequent miscarriage in RSA patients, while LA serves as an independent predictor of both thrombosis and adverse obstetric outcomes. Compared with classical APS antibodies, aPS/PT IgG and IgM are frequently detected in serologically confirmed APS cases. Supporting evidence from Hoxha et al. (22) linked aPS/PT IgG/IgM to thrombosis, pregnancy complications, and microvascular pathology, while Pleguezuelo et al. (23) reported that aPS/PT was more prevalent than aCL, anti-β_2_, or LA among women with recurrent miscarriage. Given its robust association with obstetric thrombotic complications, aPS/PT represents one of the most promising candidates aPLs for incorporation into routine RSA screening. In this study, we selected aCL, LA, and aPS/PT as differentially significant markers, and their composite demonstrated substantial predictive value for miscarriage risk, underscoring the utility of expanding current diagnostic algorithms for acquired thrombophilia in RSA.

Genetic thrombophilia ranked second in predictive importance, reflecting an individual’s inherited predisposition to thrombosis—a risk amplified by the physiological hypercoagulability of pregnancy. In the Han Chinese population, deficiencies in PC, PS, and AT-III represent the predominant genetic markers. Although a direct causal relationship with placenta-mediated complications remains debated, accumulating evidence supports its contributory role. A meta-analysis of 31 case-control, cohort, and cross-sectional studies (24) linked PS deficiency to an increased risk of late non-recurrent fetal loss (OR 7.39, 95% CI: 1.28–42.6), but not early recurrent miscarriage. The European Prospective Cohort on Thrombophilia (EPCOT) (25) further demonstrated elevated fetal loss rates among 843 women with inherited thrombophilia, reporting early miscarriage and late stillbirth in 29.4% and 23.5% of pregnancies, respectively. In our study, the combined assessment of PC, PS, and AT-III formed a robust, population-specific model for genetic thrombophilia, demonstrating strong predictive utility for RSA risk screening and supporting its integration into precision medicine frameworks for Han Chinese women.

Within our multidimensional framework, immunological factors constitute a key component in RSA risk stratification, underscoring immunological dysregulation as a key etiological driver in RSA. Autoimmune abnormality is one of the important causes of RSA, accounting for approximately 30% of all RSA causes. ANA is highly sensitive to autoimmune processes in the body and is a common indicator of autoimmune responses. Many studies have shown that ANA is correlated with RSA. Meta-analysis based on case-control studies shows that the positive rate of ANA in RSA patients is significantly higher than that in healthy pregnant women (26); Meta-analyses based on retrospective cohort studies also indicated that ANA-positive patients had a higher risk of RSA compared to ANA-negative patients (27). Evidence-based medical evidence indicates that ANA is an independent risk factor for RSA, regardless of whether it is diagnosed with other connective tissue diseases (26, 27). In addition, studies have found that abnormal maternal coagulation is also closely related to ANA antibodies. Positive ANA affects hemodynamics, leading to the formation of microthrombi in placental blood vessels. These findings underscore the importance of immunological screening in RSA evaluation—particularly in patients without overt clinical manifestations—where comprehensive immunological assessment may substantially improve diagnostic accuracy (28–30).

The coagulation profile offers a functional assessment of hemostatic balance and aids in identifying the prethrombotic state (PTS). When RSA patients are in a prethrombotic state, their coagulation function will change in the same way. When microthrombosis forms in the body accompanied by hyperfibrinolysis, it is manifested as prolonged TT. Therefore, attention should be paid to the monitoring of TT in RSA patients in clinical practice. It is more accurate to comprehensively evaluate the coagulation function by combining various causes of thrombolytic syndrome. Demographic (e.g., age, BMI) and endocrine-metabolic factors (e.g., TSH, T4) add foundational physiological context, enhancing model stability and generalizability. Collectively, our findings support a multidimensional, hierarchically weighted approach to RSA risk prediction, with acquired thrombophilia, immunological dysregulation, and genetic thrombophilia as primary drivers—enabling rational, tiered screening and optimized prenatal management, particularly in resource-limited settings.

Furthermore, we systematically refined 14 candidate predictors across six key physiological dimensions of RSA and established a simplified model comprising six core indicators: aPS/PT, AT-III, PC, ANA, TT, and BMI. This integrated panel significantly reduced testing complexity while maintaining excellent predictive performance (AUC = 0.925), offering a clinically practical strategy for precise RSA risk stratification. Among these, aPS/PT emerged as the most important marker of acquired thrombophilia. Although current APS diagnostic criteria are limited to aCL, anti-β_2_, and LA, a considerable proportion of RSA patients present as “seronegative APS” (SNAPS), manifesting typical obstetric complications despite negative classical aPLs. Recent studies (31) have shown that aPS/PT is strongly associated with early miscarriage, late fetal death, and preterm delivery, with Zigon et al. (32) reporting its higher prevalence and predictive value in OAPS compared with classical aPLs, and Zhang et al. (33) demonstrating its diagnostic utility in Chinese SNAPS cohorts. These findings underscore aPS/PT as an independent risk factor for OAPS-related pregnancy loss and a promising candidate for inclusion in revised diagnostic algorithms. Hereditary thrombophilia markers AT-III and PC were also retained in the final model, reflecting their strong contribution to hypercoagulability and adverse pregnancy outcomes. Deficiencies in AT-III, PC, and PS are among the most common hereditary thrombophilias in Asian populations, with AT-III deficiency carrying the highest risk for late fetal loss (OR 5.2, 95% CI: 1.5–18.1) and strongly associated with placental microthrombosis when activity falls below 70%. By contrast, PC and PS deficiencies generally confer lower risk individually but exert synergistic effects when combined, amplifying hypercoagulability and pregnancy complications. The inclusion of AT-III and PC in our model therefore reflects both their pathophysiological significance and predictive strength. ANA positivity was selected as the immune marker, highlighting the role of autoimmune dysregulation in RSA. High-titer ANA can drive immune complex deposition in placental vasculature, complement activation, endothelial injury, and a prothrombotic inflammatory state, consistent with its reported strong association with RSA (OR 3.0–4.0). TT shows a high predictive performance in the assessment of the pre-thrombotic state in patients with RSA. The prolongation of TT reflects the changes in coagulation function in the body, evolving from microthrombosis to hyperfibrinolysis. This pathological state may affect the impaired perfusion of the uterus and placenta. Finally, elevated BMI, the only demographic/metabolic marker in the model, independently increases RSA risk by inducing oxidative stress, low-grade inflammation, and insulin resistance, while often coexisting with conditions such as PCOS.

Collectively, these six indicators form a multidimensional predictive model that encapsulates the “coagulation–immune–metabolic” interplay underlying RSA. Their broad coverage clinical dimensions emphasizes that RSA arises from synergistic, multisystem perturbations, rather than isolated defects. Importantly, SHAP analyses confirmed that even when individual markers remain within normal ranges, their combined patterns can reveal high-risk phenotypes consistent with a multifactorial pathogenic model. For example, we found that the combination of biomarkers such as aPS/PT positivity, prolonged TT, and AT-III deficiency had a significant impact on coagulation function. Among these, the combinations of aPS/PT + TT, AT-III + aPS/PT, and TT + PC showed a strong synergistic effect, revealing the combined effect of immune and abnormal coagulation functions. These characteristic combinations, especially when immune abnormalities and coagulation dysfunction coexist, form a “double blow” state, leading to a significant increase in the risk of miscarriage. The combinations of aPS/PT + PC, ANA + PC, BMI + ANA, AT-III + TT, and TT + BMI showed characteristics of high risk in miscarriage. These combinations fully demonstrated the synergistic effect of immune factors and coagulation functions, increasing the risk of miscarriage. Moreover, patients with overweight or obesity have a chronic inflammatory state, with increased inflammatory factors and excessive oxidative stress response. When these factors accumulate with the synergistic effect of immune and coagulation factors, the risk of miscarriage increases significantly. It is worth noting that in demographic characteristics, abnormal BMI (>24) as a diagnostic indicator of overweight, combined with immune factors (ANA) and coagulation factors (TT), becomes a medium to high-risk factor for miscarriage, further emphasizing the complexity of the synergistic effect of immune and coagulation factors and the aggravation of the risk of miscarriage. Such synergistic effects illustrate the complementary information captured by integrating multiple clinical domains, enabling identification of otherwise “hidden high-risk” individuals. This paradigm shift—from isolated marker testing to system-level composite modeling—provides a robust framework for precision screening and early intervention in RSA, demonstrating that the strategic synthesis of routine laboratory data can effectively capture the pathogenic complexity typically sought in systemic biological evaluations.

Despite these significant findings, several limitations must be acknowledged. First, all data were collected from a single center, which may introduce selection bias and limit the external validity of the model. Although this study successfully validated the model’s ability to distinguish high-risk RSA patients from healthy controls, its performance has not yet been assessed in an unselected, general population cohort. Future research should therefore conduct prospective validation in such unselected cohorts to calibrate the tool for broader screening applications in the general population. Additionally, while several key predictors were identified, the complex interaction mechanisms among them warrant deeper investigation; integrating multidimensional data and incorporating molecular biology approaches will be essential to further elucidate the pathophysiological mechanisms underlying RSA and to support the development of more accurate predictive tools and targeted therapeutic strategies.

In conclusion, this study presents a highly accurate predictive model for RSA and provides novel insights into the complex, multi-system interactions underlying its etiology. A six-biomarker panel was identified for practical, cost-effective population screening. These findings hold promise for advancing personalized approaches to RSA prevention and management, ultimately improving reproductive health outcomes for affected individuals.

## Data Availability

The raw data supporting the conclusions of this article will be made available by the authors, without undue reservation.
