# Supplementary material for: A multidimensional clinical prediction model for early screening of recurrent spontaneous abortion: integrating coagulation, immune, and endocrine markers
Source: Front Immunol. 2026 Mar 6;17:1774359. doi: 10.3389/fimmu.2026.1774359 (PMC13002355; doi:10.3389/fimmu.2026.1774359)
Supplement: Supplementary file 1 [file Table1.docx]

Supplementary Material

# Supplementary Figures and Tables

## Supplementary Figures

**
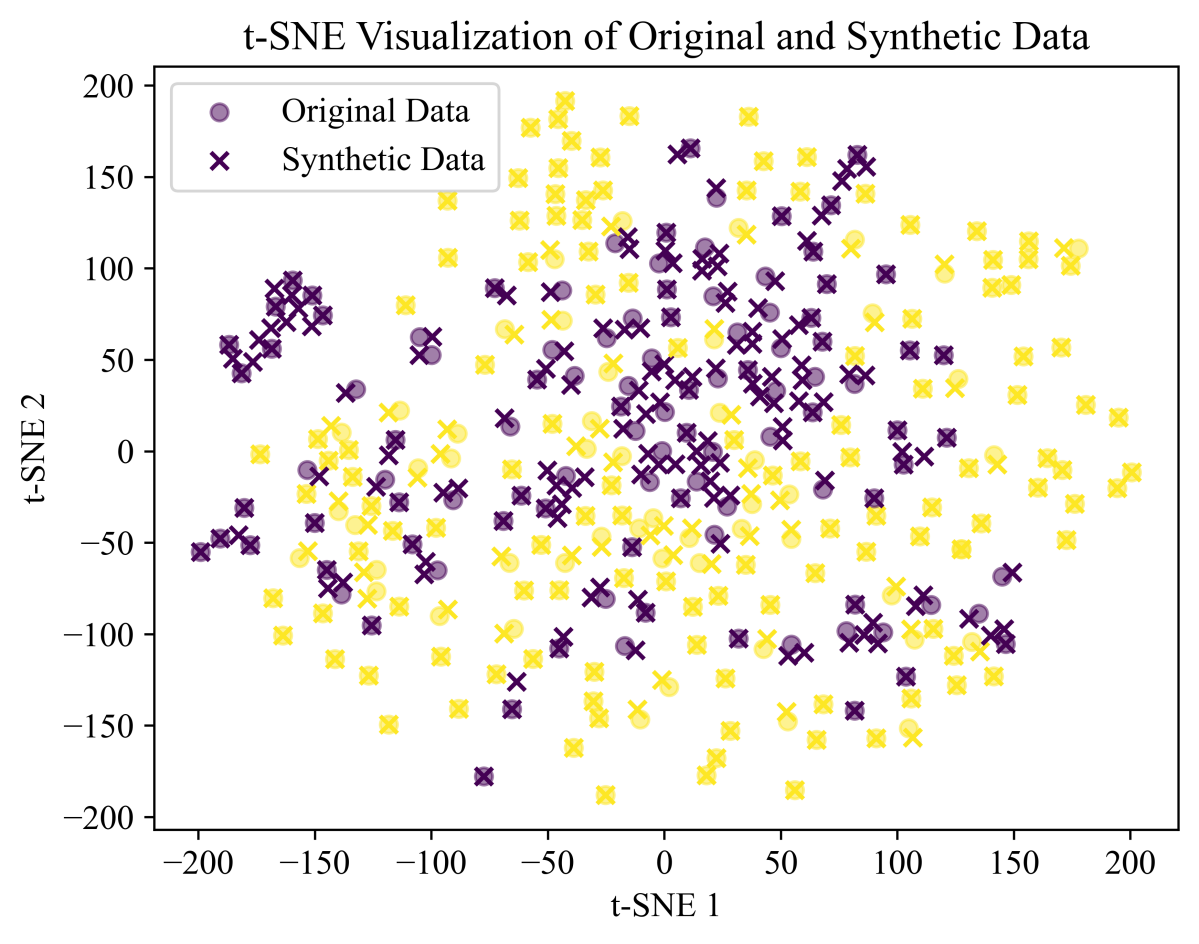
**

**Figure S1. Sample distribution map after SMOTE data augmentation.** The number of fewer samples was increased, alleviating the problem of sample imbalance. "t-SNE 1" and "t-SNE 2" are two feature dimensions obtained after reducing the dimension of the data through the t-SNE (T-Distributed Neighborhood Embedding) algorithm.


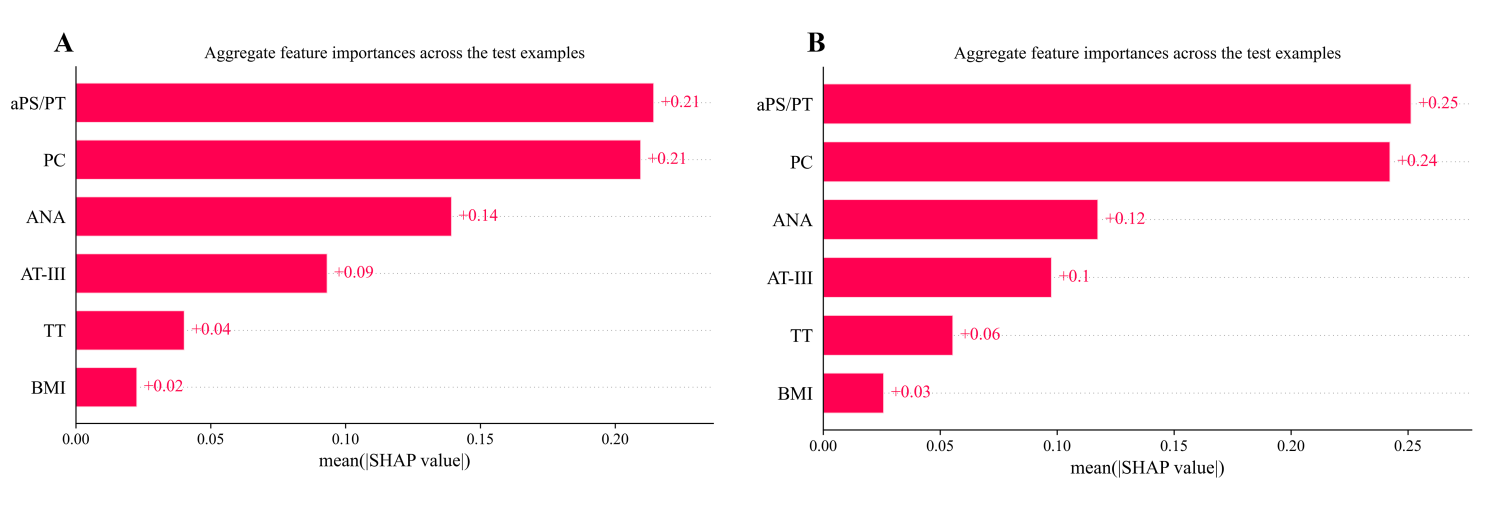


**Figure S2. SHAP Feature Consistency Analysis.** (A) Base model SHAP feature importance; (B)Aug model SHAP feature importance.


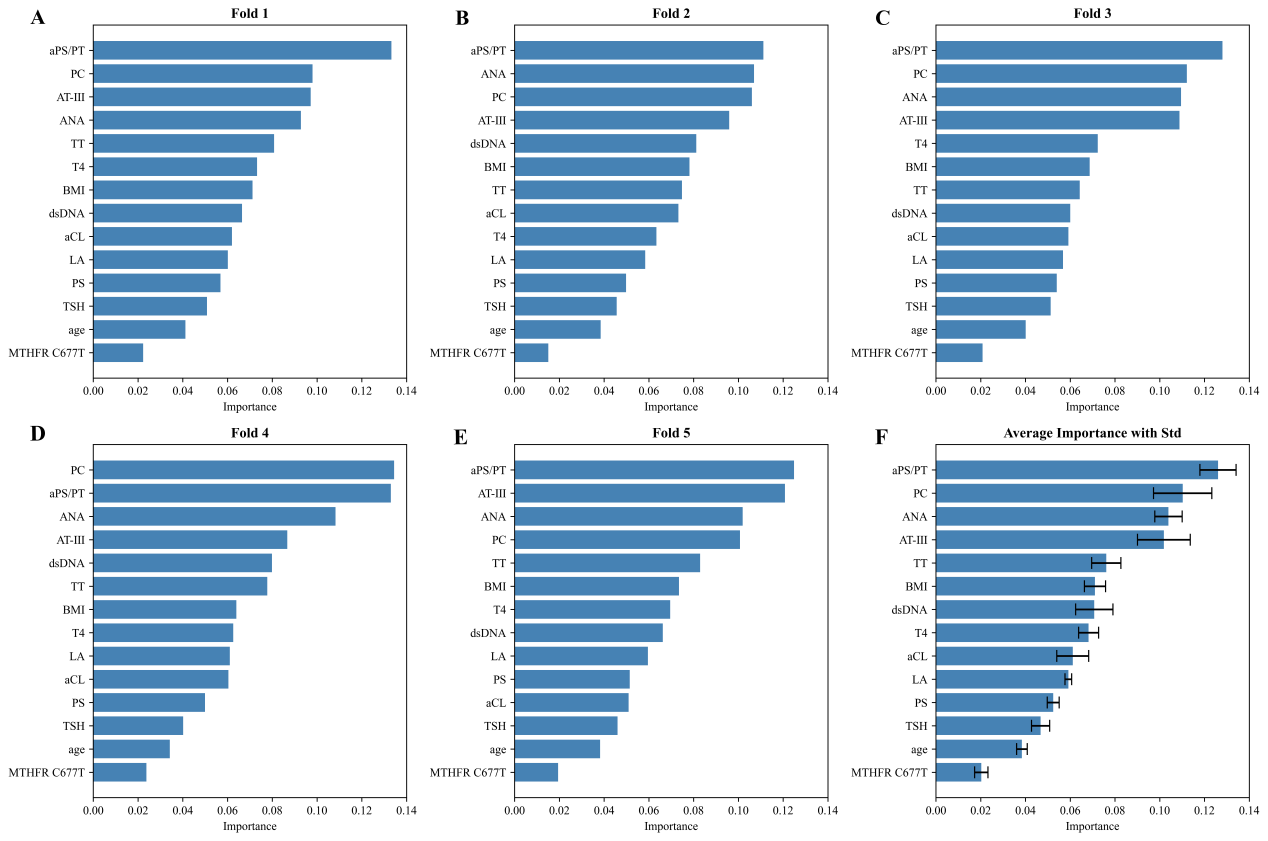


**Figure S3. Feature importance rankings across five cross-validation folds based on Mean Decrease in Impurity (MDI) from Random Forest models.** (A–E) The MDI values of each feature within individual training folds (Fold 1 to Fold 5), computed independently using only the training data to prevent data leakage. (F) The average MDI importance across all folds, with error bars representing standard deviation. The six features—aPS/PT, PC, ANA, AT-III, TT, and BMI—exhibited the highest mean importance scores across folds, forming the basis of the final parsimonious predictive panel.

**
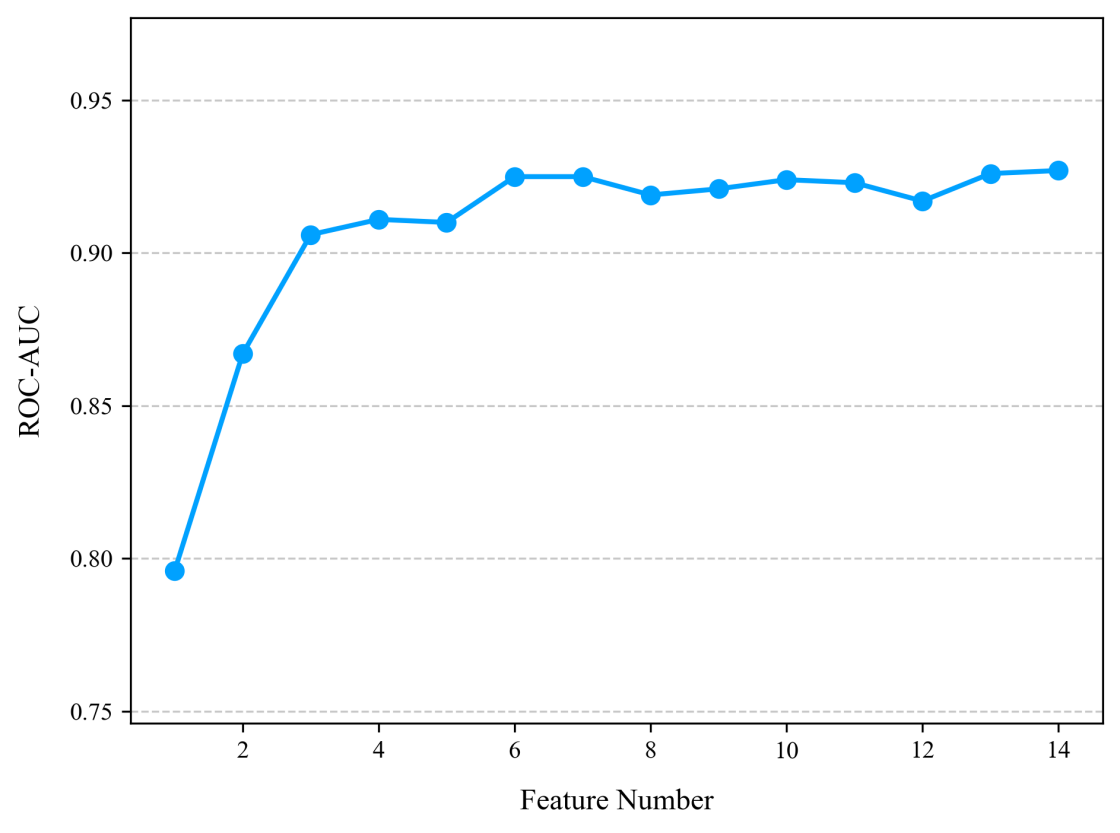
**

**Figure S4. The trend line graph of ROC-AUC values eliminated by recursive features.** The horizontal axis representing the number of features and the vertical axis representing the ROC-AUC values.


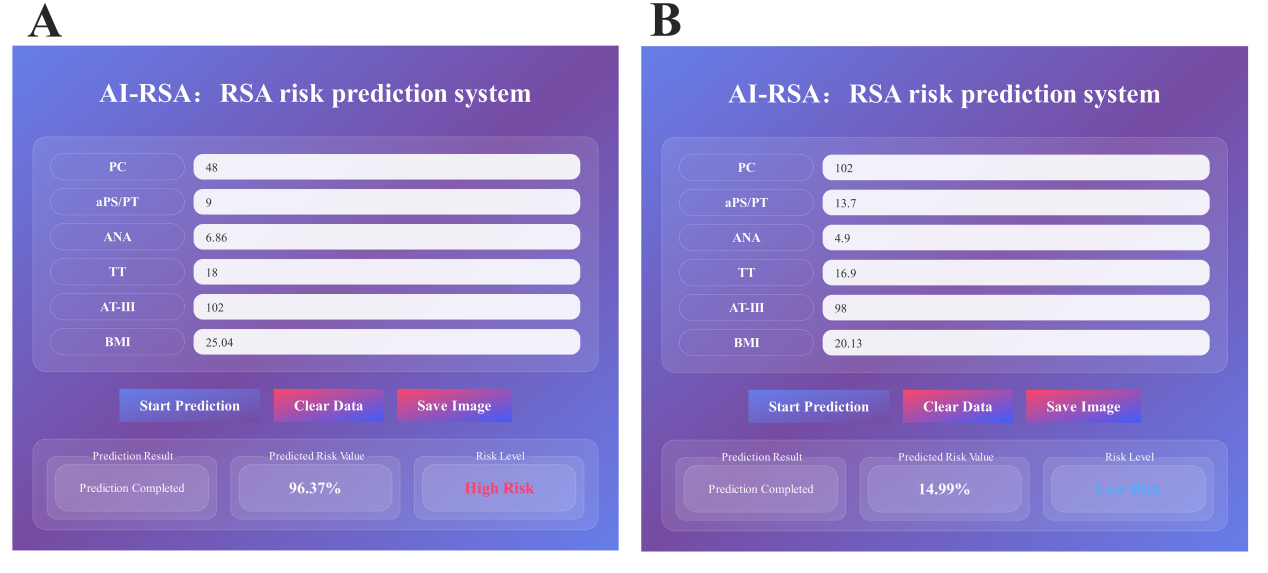


**Figure S5. Clinicians input the values of six indicators (aPS/PT, PC, ANA, AT-III, TT, BMI) in the AI-RSA platform, and the platform outputs the disease risk level:** (A) High Risk; (B) Low Risk.

- 1. **Supplementary Tables**

**Table S1.** Parameter settings for all classifiers.

| **Classifier** | **Parameter Settings** |
| --- | --- |
| DT | Maximum depth = 10; Pre - pruning strategy; Feature splitting based on information gain / Gini index |
| KNN | Number of neighbors = 5; Distance metric = Euclidean distance; Voting strategy = Majority voting |
| MLP | Hidden layer size = (10,); Activation function = ReLU; Output layer = Sigmoid; Maximum number of iterations = 1000; Dropout = 0.2 |
| NB | Distribution assumption = Gaussian distribution; Based on the feature conditional independence assumption |
| LR | Maximum number of iterations = 1000; L2 regularization; Optimization algorithm = Adam |
| SVM | Kernel function = poly; C = 10.0; gamma = auto |
| RF | Number of trees = 100; Maximum depth = 5; Random selection of feature sub - spaces; Feature importance calculated based on MDI |
| GB | Number of trees = 100; Learning rate = 0.1; Maximum depth = 3; Loss function = log_loss |
| AB | Number of boosting iterations = 100; Base learner = Decision tree (depth = 1); Dynamic adjustment of sample weights |
| XGBoost | Number of trees = 100; Learning rate = 0.1; Maximum depth = 3; Sub - sampling rate = 0.8; Column sampling rate = 0.7; scale_pos_weight = 2.9 |
| TabPFN | n_estimators = 4; softmax_temperature = 0.8 |

**Table S2.** Parameter settings for TabPFN

| **Parameter Category** | **Parameter Name** | **Value** | **Notes** |
| --- | --- | --- | --- |
| Pre-trained Architecture | Transformer Layers | 12 | Encoder-only Transformer layers |
|  | Attention Heads | 4 | Multi-head attention mechanism |
|  | Embedding Dim | 512 | Input feature embedding dimension |
|  | FFN Dim | 1024 | Feed-forward network hidden dimension |
|  | Learning Rate Schedule | Cosine annealing | Default strategy in tabpfn v0.1.9 |
|  | Early Stopping Criteria | Val-AUC no improvement for 5 epochs | Encapsulated in pre-training pipeline |
| Inference Settings | n_estimators | 4 | Ensemble of 4 base models |
|  | softmax_temperature | 0.8 | Probability calibration parameter |
| Computational Environment | Hardware | Nvidia RTX4060 (8GB) | GPU acceleration for inference |
|  | Software | tabpfn library v0.1.9 | [Open-source library ( https://github.com/automl/TabPFN )](https://github.com/automl/TabPFN) |

**Table S3.** The effect of the SMOTE data augmentation.

| **Classifier** | **Accuracy** | **Precision** | **Sensitivity** | **Specificity** | **F1 Score** | **ROC-AUC** |
| --- | --- | --- | --- | --- | --- | --- |
| Base model  (train) | 0.845 | 0.921 | 0.810 | 0.866 | 0.861 | 0.929 |
| Base model  (test) | 0.826 | 0.904 | 0.804 | 0.856 | 0.850 | 0.926 |
| Base model gap | 0.019 | 0.017 | 0.006 | 0.010 | 0.011 | 0.003 |
| Aug model  （train） | 0.867 | 0.931 | 0.852 | 0.875 | 0.889 | 0.933 |
| Aug model  (test) | 0.846 | 0.908 | 0.840 | 0.858 | 0.872 | 0.927 |
| Aug model gap | 0.021 | 0.023 | 0.012 | 0.017 | 0.017 | 0.006 |

**Table S4.** Stability of SHAP-based Feature Importance Across 5 Folds

| **Feature** | **Mean(\|SHAP\|)** | **SD** | **CV** | **Stability** |
| --- | --- | --- | --- | --- |
| aPS/PT | 0.248 | 0.029 | 0.117 | High |
| PC | 0.215 | 0.031 | 0.146 | High |
| ANA | 0.118 | 0.014 | 0.123 | High |
| AT-III | 0.111 | 0.015 | 0.132 | High |
| TT | 0.066 | 0.009 | 0.14 | High |
| BMI | 0.034 | 0.014 | 0.397 | Moderate |

**Table S5.** Performance comparison of classification algorithms (Mean ± Standard Deviation).

| **Classifier** | **Accuracy** | **Precision** | **Recall** | **Specificity** | **F1 Score** | **ROC AUC** |
| --- | --- | --- | --- | --- | --- | --- |
| MLP | 0.676±0.053 | 0.768±0.046 | 0.690±0.055 | 0.655±0.068 | 0.725±0.048 | 0.724±0.040 |
| DT | 0.734±0.039 | 0.807±0.052 | 0.755±0.056 | 0.701±0.108 | 0.778±0.030 | 0.725±0.061 |
| LR | 0.680±0.066 | 0.783±0.057 | 0.672±0.106 | 0.694±0.101 | 0.720±0.076 | 0.743±0.071 |
| SVM | 0.520±0.079 | 0.889±0.071 | 0.263±0.081 | 0.800±0.133 | 0.394±0.068 | 0.756±0.062 |
| KNN | 0.691±0.045 | 0.831±0.060 | 0.632±0.059 | 0.789±0.084 | 0.716±0.049 | 0.785±0.065 |
| NB | 0.662±0.056 | 0.812±0.056 | 0.609±0.079 | 0.813±0.057 | 0.696±0.068 | 0.791±0.030 |
| AdBoost | 0.807±0.051 | 0.847±0.078 | 0.848±0.052 | 0.741±0.158 | 0.847±0.035 | 0.865±0.029 |
| XGB | 0.821±0.038 | 0.840±0.049 | 0.883±0.054 | 0.721±0.101 | 0.860±0.030 | 0.881±0.027 |
| RF | 0.815±0.021 | 0.846±0.018 | 0.860±0.045 | 0.741±0.040 | 0.852±0.020 | 0.896±0.022 |
| GB | 0.840±0.034 | 0.861±0.059 | 0.889±0.064 | 0.760±0.109 | 0.874±0.027 | 0.897±0.036 |
| TabPFN | 0.826±0.042 | 0.904±0.015 | 0.804±0.079 | 0.856±0.032 | 0.850±0.038 | 0.926±0.015 |

**Table S6.** The effect of the SMOTE data augmentation (Mean ± Standard Deviation).

| **Classifier** | **Accuracy** | **Precision** | **Recall** | **Specificity** | **F1 Score** | **ROC AUC** |
| --- | --- | --- | --- | --- | --- | --- |
| Base model | 0.826±0.042 | 0.904±0.015 | 0.804±0.079 | 0.856±0.032 | 0.850±0.038 | 0.926±0.015 |
| Aug model | 0.846±0.022 | 0.908±0.027 | 0.84±0.067 | 0.858±0.058 | 0.872±0.024 | 0.927±0.012 |

**Table S7.** Ablation study of six key physiological dimensions features sets (Mean ± Standard Deviation).

| **Features** | **Accuracy** | **Precision** | **Recall** | **Specificity** | **F1 Score** | **ROC AUC** |
| --- | --- | --- | --- | --- | --- | --- |
| ALL | 0.846±0.022 | 0.908±0.027 | 0.84±0.067 | 0.858±0.058 | 0.872±0.024 | 0.927±0.012 |
| Without A | 0.758±0.058 | 0.860±0.052 | 0.732±0.105 | 0.800±0.09 | 0.790±0.064 | 0.856±0.023 |
| Without H | 0.756±0.05 | 0.846±0.076 | 0.742±0.034 | 0.768±0.136 | 0.790±0.036 | 0.862±0.029 |
| Without I | 0.810±0.049 | 0.876±0.04 | 0.812±0.062 | 0.808±0.067 | 0.842±0.043 | 0.876±0.031 |
| Without C | 0.820±0.041 | 0.896±0.027 | 0.806±0.059 | 0.846±0.038 | 0.848±0.038 | 0.914±0.036 |
| Without D | 0.844±0.029 | 0.904±0.028 | 0.840±0.08 | 0.846±0.061 | 0.870±0.032 | 0.922±0.015 |
| Without E | 0.844±0.032 | 0.892±0.031 | 0.852±0.059 | 0.828±0.054 | 0.870±0.029 | 0.922±0.008 |

**Table S8.** Model performance during RFE (Mean ± Standard Deviation).

| **Number of Feature** | **Accuracy** | **Precision** | **Recall** | **Specificity** | **F1 Score** | **ROC AUC** |
| --- | --- | --- | --- | --- | --- | --- |
| 14 | 0.846±0.022 | 0.908±0.027 | 0.84±0.067 | 0.858±0.058 | 0.872±0.024 | 0.927±0.012 |
| 13 | 0.840±0.027 | 0.901±0.029 | 0.836±0.061 | 0.847±0.055 | 0.866±0.027 | 0.926±0.013 |
| 12 | 0.840±0.037 | 0.900±0.021 | 0.836±0.066 | 0.846±0.035 | 0.865±0.036 | 0.917±0.014 |
| 11 | 0.833±0.029 | 0.899±0.022 | 0.825±0.064 | 0.847±0.046 | 0.859±0.030 | 0.923±0.010 |
| 10 | 0.836±0.041 | 0.894±0.023 | 0.836±0.071 | 0.837±0.036 | 0.863±0.038 | 0.924±0.013 |
| 9 | 0.836±0.041 | 0.910±0.018 | 0.819±0.082 | 0.866±0.035 | 0.860±0.040 | 0.921±0.020 |
| 8 | 0.836±0.034 | 0.901±0.034 | 0.830±0.065 | 0.846±0.055 | 0.862±0.033 | 0.919±0.016 |
| 7 | 0.847±0.036 | 0.901±0.018 | 0.848±0.068 | 0.846±0.035 | 0.872±0.033 | 0.925±0.018 |
| 6 | 0.833±0.047 | 0.911±0.031 | 0.813±0.090 | 0.865±0.056 | 0.856±0.044 | 0.925±0.018 |
| 5 | 0.829±0.055 | 0.904±0.038 | 0.813±0.083 | 0.855±0.062 | 0.854±0.049 | 0.910±0.019 |
| 4 | 0.785±0.018 | 0.910±0.040 | 0.731±0.060 | 0.876±0.064 | 0.808±0.023 | 0.911±0.024 |
| 3 | 0.789±0.039 | 0.895±0.056 | 0.754±0.068 | 0.847±0.092 | 0.815±0.037 | 0.906±0.031 |
| 2 | 0.753±0.059 | 0.871±0.053 | 0.708±0.067 | 0.827±0.072 | 0.780±0.057 | 0.867±0.040 |
| 1 | 0.698±0.045 | 0.921±0.070 | 0.567±0.058 | 0.913±0.076 | 0.699±0.051 | 0.796±0.072 |

**Table S9.** The model performance of different population subgroups (Mean ± Standard Deviation)

| **Classifier** | **Accuracy** | **Precision** | **Recall** | **Specificity** | **F1 Score** | **ROC AUC** |
| --- | --- | --- | --- | --- | --- | --- |
| All people | 0.833±0.047 | 0.911±0.031 | 0.813±0.090 | 0.865±0.056 | 0.856±0.044 | 0.925±0.018 |
| High Age | 0.881±0.044 | 0.919±0.028 | 0.913±0.035 | 0.843±0.041 | 0.915±0.033 | 0.933±0.016 |
| High BMI | 0.943±0.039 | 0.951±0.027 | 0.921±0.022 | 0.936±0.039 | 0.935±0.058 | 0.947±0.011 |

**Note 1: Clinical Workflow and Implementation Guardrails**

**Hardware and Latency:**

The reported inference time (5 ms) refers to the computational processing of the TabPFN model on a standard central processing unit (CPU). Including manual data entry and automated preprocessing, the total response time is negligible (<1 second), enabling real-time usage during patient visits.

**Intended Clinical Workflow:**

The platform functions as a screening and risk stratification tool rather than a standalone diagnostic device.

1. **Input:** Obstetricians enter the six required clinical parameters during early pregnancy consultations.
2. **Output:** The model generates a probability score for recurrent spontaneous abortion (RSA).
3. **Action:** High-risk predictions are protocolized to trigger enhanced surveillance (e.g., increased frequency of ultrasound monitoring, hormonal profiling) rather than immediate irreversible interventions.

**Guardrails and Safety Considerations:**

In the context of RSA management, the clinical priority favors sensitivity to prevent the devastating consequences of missed high-risk cases (False Negatives). Consequently, a False Positive result (overestimation of risk) leads to “protective” management strategies. To mitigate potential patient anxiety and unnecessary treatment:

- **Physician Oversight:** All outputs are labeled as “Screening Results” to emphasize that clinical judgment remains the final arbiter. High-risk flags require a thorough clinical review and confirmatory testing before initiating pharmacological interventions (e.g., progesterone support).
